# The Italian version of the unified theory of acceptance and use of technology questionnaire: a pilot validation study

**DOI:** 10.3389/frobt.2025.1371583

**Published:** 2025-02-17

**Authors:** Alfonsina D’Iorio, Federica Garramone, Silvia Rossi, Chiara Baiano, Gabriella Santangelo

**Affiliations:** ^1^ Department of Psychology, University of Campania “Luigi Vanvitelli”, Caserta, Italy; ^2^ Department of Electrical Engineering and Information Technologies, University of Naples Federico II, Napoli, Italy

**Keywords:** UTAUT, SAR, technology acceptance, robotics technology, validity, reliability

## Abstract

**Background:**

The Unified Theory of Acceptance and Use of Technology is a self-rated questionnaire to assess twelve constructs related to the level of acceptance of a robot, consisting of 41 items rated on a 5-point Likert scale. The aim of the study was to conduct a preliminary evaluation of the psychometric properties of the Italian version of the UTAUT (I-UTAUT) in a sample of Italian healthy subjects (HCs).

**Materials and methods:**

30 HCs underwent the I-UTAUT to assess its comprehensibility. Reliability and divergent validity of the I-UTAUT were evaluated in a sample of 121 HCs, who also underwent the Montreal Cognitive Assessment (MoCA).

**Results:**

The final I-UTAUT version was easily comprehensible. There were no missing data, no floor and ceiling effects. Contrarily to the original version, the Principal Components Analysis suggested a seven-component structure; Cronbach’s alpha was 0.94. The I-UTAUT score did not correlate with MoCA.

**Conclusion:**

The I-UTAUT represented a reliable and valid questionnaire to identify the level of acceptance of robotics technology in Italian healthy sample.

## Introduction

The number of older people living alone and in need of care has grown during the last decades and the scientific research is increasingly providing evidence of the possible successful application of the Social Assistive Robotics (SAR) in supporting the elderly users. In detail, SAR represent a class of technological devices that combines social interaction with assistive technology to support individuals, particularly the elderly or individuals requiring assistance in daily activities.

Accordingly, recent studies (Cavallo et al., 2018; Di Nuovo et al., 2016; Di Nuovo et al., 2018; Olaronke et al., 2017) showed that SAR can be successfully employed in healthcare environments due to their peculiarity of being able to provide personalized treatments making use of adaptable social skills. Another study (van Dam et al., 2022) showed encouraging results about the use of SAR in increasing the independence of people with executive dysfunctions in disability care.

However, despite the growing interest in developing this type of technology for supporting older people, one-third of all assistive technologies are abandoned within 1 year of use (Gurley and Norcio, 2009) because of fear of trying something new, the lack of training to use new technologies or not perceiving the need for technology (Bevilacqua et al., 2014; Lattanzio et al., 2014; Tacken et al., 2005). For this reason, one of the most important goals of robotics is to be able to give the robot the highest degree of acceptability.

Robot acceptance is influenced by three primary factors (Beer et al., 2011): the robot’s functionality, social abilities, and appearance. Functionality encompasses the robot’s potential role in domestic settings, particularly concerning tasks pertinent to daily living and healthcare, especially for individuals with physical limitations or older adults. A significant aspect of functionality is the level of autonomy, which ranges from full human control (teleoperation) to fully autonomous robot control. Aligning the autonomy level with user expectations is crucial for enhancing acceptance (Huang et al., 2005).

The manner in which humans’ control and interface with robots also plays an essential role in acceptance. When designing robots, it is critical to consider the appropriateness of control methods for specific tasks alongside user preferences and ease of use (Scholtz, 2003).

Social abilities, particularly social intelligence, prominently affect how robots interact with humans. Emotion-expressive capabilities, such as the ability to convey facial expressions, are fundamental for creating advanced intelligent robotic agents, as they can respond sensitively to human emotions and social cues (Cassell et al., 2000). Non-verbal cues, including gestures like nodding and eye movements, also significantly impact interactions, helping to establish a collaborative relationship between humans and robots (Bartneck et al., 2004).

The robot’s appearance further influences acceptance. To facilitate positive interactions, robot design should ensure that users can easily interpret the robot’s behavior (Kanda et al., 2008).

To better understand how various robot-related aspects impact users’ acceptance of robotic systems, several technology acceptance models have been proposed. A notable framework in this context is the Unified Theory of Acceptance and Use of Technology (UTAUT), which was formulated in 2003 by Venkatesh et al. (2003) with the aim to develop a unified model of the eight prominent models of users’ technology acceptance (i.e., the theory of reasoned action, the technology acceptance model, the motivational model, the theory of planned behavior, a model combining the technology acceptance model and the theory of planned behavior, the model of PC utilization, the innovation diffusion theory and the social cognitive theory). Overall, the UTAUT model has been widely used in research to understand how and why individuals and organizations adopt and utilize new technologies, providing valuable insights for designing systems that encourage acceptance and effective use.

Heerink et al. (2009) developed a questionnaire based on the UTAUT model in order to provide robotics developers with a straightforward instrument able to evaluate users’ acceptance of SAR designing or developing for elderly care environments.

In light of the abovementioned issues, it is clear the need of specific and validated instruments which could help human-robot interaction developers in evaluating the acceptability of their own robots and increasing their usability according to users’ attitudes and preferences.

Therefore, the aim of the present study was to conduct a preliminary evaluation of the reliability and validity of the Italian version of the UTAUT questionnaire (I-UTAUT) in a sample of Italian healthy subjects (HCs).

## Materials and methods

### Italian adaptation of the UTAUT questionnaire

The UTAUT, a self-rated questionnaire, consists of 41 items rated on a 5-point Likert scale. The original version of the scale investigates thirteen constructs related to the level of acceptance of a robot. In particular: Anxiety (ANX): Evoking anxious or emotional reactions when using the system; Attitude (ATT): Positive or negative feelings about the appliance of the technology; Facilitating conditions (FC): Objective factors in the environment that facilitate using the system; Intention to use (ITU): The outspoken intention to use the system over a longer period in time; Perceived adaptability (PAD): The perceived ability of the system to be adaptive to the changing needs of the user; Perceived Enjoyment (PENJ) Feelings of joy or pleasure associated by the user with the use of the system; Perceived ease of use (PEOU): The degree to which the user believes that using the system would be free of effort; Perceived sociability (PS): The perceived ability of the system to perform sociable behavior; Perceived Usefulness (PU): The degree to which a person believes that using the system would enhance his or her daily activities; Social Influence (SI): The user’s perception of how people who are important to him think about him using the system; Social Presence (SP): The experience of sensing a social entity when interacting with the system; Trust (Trust): The belief that the system performs with personal integrity and reliability and finally Use/Usage (USE): The actual use of the system over a longer period in time.

We adapted the UTAUT to Italian language by a translation/re-translation method and, compared to the original version of the questionnaire, we also added preliminary questions aimed to explore the subjects’ perceived familiarity with the use of the technology. Two independent researchers translated the item of the questionnaire and its accompanying instructions. Subsequently, the two translations were merged into a draft and all discrepancies were examined in a consensus meeting. Then, the questionnaire was translated back to English according to Beaton et al. (2000) guidelines (2000) by an independent and English mother tongue translator with no knowledge of the questionnaire.

A panel of independent experts compared the back-translated and the original version of UTAUT to evaluate the linguistic equivalence of the two English versions and approve the translation.

Finally, to explore the appropriateness and the comprehensibility of the provisional Italian translation, an examiner administrated the Italian version of the UTAUT to a group of 30 healthy subjects (HCs) aged from 30 to >80 years. In detail, participants were asked to evaluate the comprehensibility of the instructions and response options responding “Yes” (coded 1) if they were easily understood or “No” (coded 0) if they were poorly understood.

All items and instructions were judged as easily understood by >95% of participants; thus, the Italian version of the UTAUT was considered as final. A copy of the I-UTAUT is available in the Supplementary Material S1.

### Population

The I-UTAUT was administrated to consecutive HCs, recruited among employees and in elderly clubs. To be included in the study, subjects had to meet the following inclusion criteria: i. lack of general cognitive decline, as defined by a normal age- and education-adjusted score on the Italian version of the Montreal Cognitive Assessment (total age and education adjusted score on MoCA ≥15.5) (Santangelo et al., 2015); ii, no history of neurological and/or psychiatric disorders based on the DSM-V criteria; iii, being native Italian-speaking.

All participants gave their written informed consent; the study was approved by the local ethics committee.

### Statistical analysis

Acceptability of the I-UTAUT was considered appropriate if there was <5% of missing values and <15% of the respondents with the lowest or highest scores (floor and ceiling effects), and a skewness ranging −2 to +2.

Principal Components Analysis (PCA) with Promax Oblique rotation was used to explore the factorial structure of the scale. Following recent guidelines, a sample size of N = 100 is deemed as sufficient to perform a PCA (Kyriazos, 2018). PCA assumptions were tested via Bartlett’s sphericity test and the Kaiser-Meyer-Olkin (KMO) statistic (this last measure being judged as acceptable if >0.6). Items that did not meet the loading threshold of ≥0.4 on any component were considered insufficiently associated with the primary constructs and were removed from further analysis. Additionally, any item that was the sole representative of a component was also excluded, as it lacked sufficient support to form a coherent factor. These criteria ensured that retained items contributed meaningfully to the identified factors, enhancing the clarity and interpretability of the component structure. Then, we conducted a pilot Structural Equation Modeling (SEM) analysis with bootstrapping (1,000 repetitions) to obtain a more robust evaluation of the factor structure of the scale. To identify potentially significant parameters to be added, the modification indices (MIs) (Kline, 2011) of the tested model were also taken into account. The model that included all relevant parameters was used as the reference model. Regarding fit indices, the maximum likelihood (MLχ2) goodness-of-fit test statistics, in combination with the root mean square error of approximation (RMSEA) index and the comparative fit index (CFI) were employed. The following thresholds were considered indicative of acceptable fit: RMSEA values <0.08 and CFI values >0.90 (Browne and Cudeck, 1993).

The internal consistency was assessed using Cronbach’s alpha coefficient, item-total correlation and the average inter-item correlation. The questionnaire was considered to have good reliability if Cronbach’s alpha was >0.70 and if item-total correlation and inter-item correlation was >0.30.

Construct validity was assessed by means of divergent validity. Therefore, correlation of the I-UTAUT score with MoCA score and its sub-domains was performed by Pearson’s correlation coefficient. Moreover, correlation between I-UTAUT and demographic features (i.e., age and years of schooling) was also calculated by Pearson correlation coefficients.

Convergent validity was not assessed because of the absence in literature of further, gold-standard standardized measures of the target construct, to be used as comparison for the proposed instrument.

All analyses were performed using SPSS version-24 (SPSS Inc., Chicago, IL) and JASP version 0.19.2.

## Results

A total of 121 HCs were enrolled (53 men and 68 women). Background and psychometric outcomes are displayed in Table 1. There was no missing data, no floor or ceiling effects.

**TABLE 1:** Descriptive statistics.

	Minimum	Maximum	Mean	Standard deviation
Age (years)	30	87	53.14	15.57
Education (years)	2	18	11.91	3.55
MoCA total score	9	29	21.85	4.43
*Visuospatial subscore*	0	4	3.12	1.07
*Attention subscore*	1	6	4.31	1.35
*Language subscore*	0	6	4.55	1.16
*Memory subscore*	0	6	2.08	1.42
*Orientation subscore*	3	6	5.88	0.49
I-UTAUT-original version				
Total score	0	194	114.26	33.32
*ANX subscore*	0	20	13.19	4.19
*ATT subscore*	0	15	9.72	3.22
*FC subscore*	0	10	4.68	1.96
*ITU subscore*	0	15	6.74	3.40
*PAD subscore*	0	15	9.52	3.10
*PENJ subscore*	0	25	16.20	5.42
*PEOU subscore*	0	25	14.62	4.37
*PS subscore*	0	20	9.48	4.20
*PU subscore*	0	15	9.63	3.23
*SI subscore*	0	10	5.64	2.32
*SP subscore*	0	23	10.30	4.29
*TRUST subscore*	0	10	4.52	2.28

I-UTAUT, Italian version of UTAUT questionnaire; MoCA, Montreal Cognitive Assessment; ANX, anxiety; ATT, Attitude; FC, Facilitating Conditions; ITU, Intention of Use; PAD, Perceived Adaptability; PENJ, Perceived Enjoyment; PEOU, Perceived Ease of Use; PS, Perceived Sociability; PU, Perceived Usefulness; SI; Social Influence; SP, Social Presence.

The PCA revealed nine eigenvalues exceeding 1, accounting for 72.5% of variance. However, items 14, 15, and 21 were removed due to low loadings across all components, indicating limited alignment with the primary constructs measured. Additionally, item 38 was removed as it loaded onto a unique factor without any supporting items, failing to provide a stable representation of any broader construct. After the removal of these items, a subsequent PCA was conducted, which revealed seven eigenvalues exceeding 1, accounting for 70% of variance. The scree-plot revealed a four-factor model. However, we chose to rely on the eigenvalues, as this approach was more consistent with the original structure of the scale.

The first component (C1) explained 21.6% of variance, and included items related to the acceptance and perceived usefulness of the robot (Acceptance and Perceived Usefulness; M = 49.25; SD = 11). The second component (C2) explained 16.8% of variance, and included items representing the perceived sociability and empathy of the robot (Perceived Sociability; M = 15.18; SD = 5.92). The third component (C3) explained 10.2% of variance, including items representing user’s individual self-efficacy and intention to use the robot (Self-Efficacy and Usage Intention; M = 11.76; SD = 4.42). The fourth component (C4) explained 0.8% of variance, and included items representing confidence and perceived ease of use of the robot (Confidence and Ease of Use; M = 11.27; SD = 3.13). The fifth component (C5) explained 0.6% of variance, and included items indexing feelings of anxiety or emotional reactions towards the robot (Anxiety; M = 7.92; SD = 1.79). The sixth component (C6) explained 0.38% of variance, and included items indexing need for support in using the robot (Need for Support; M = 6.81; SD = 1.45). The seventh component (F7) explained 0.37% of variance, and included items representing trusting the robot’s advice (Trust; M = 4.71; SD = 2.07). The factors significantly correlated with total I-UTAUT score and among each other (Table 2). The total score of the I-UTAUT was M = 106.92; SD = 21.15.

**TABLE 2:** Items loadings of the Principal Component Analysis for the I-UTAUT.

	C1	C2	C3	C4	C5	C6	C7
6. The robot would make my life more interesting	0.0884						
5. I think it’s a good idea to use the robot	0.881						
18. I find the robot enjoyable	0.869						
7. It’s good to make use of the robot	0.851						
17. I enjoy doing things with the robot	0.772						
19. I find the robot fascinating	0.732						
29. I think the robot is nice	0.676						
30. I think the robot is useful to me	0.675						
16. I enjoy the robot talking to me	0.666						
32. I think the staff would like me using the robot	0.623						
13. I think the robot can be adaptive to what I need	0.622						
31. It would be convenient for me to have the robot	0.552						
34. I think it would give a good impression if I should use the robot	0.524						
20. I find the robot boring	0.462						
33. I think the staff would like me using the robot	0.440						
28. I feel the robot understands me		0.896					
35. When interacting with the robot I felt like I’m talking to a real person		0.891					
27. I find the robot pleasant to interact with		0.867					
26. I consider the robot a pleasant conversational partner		0.818					
36. It sometimes felt as if the robot was really looking at me		0.742					
37. I can imagine the robot to be a living creature		0.729					
39. Sometimes the robot seems to have real feelings		0.678					
10. I think I’ll use the robot during the next few days			0.866				
12. I’m planning to use the robot during the next few days			0.879				
11. I am certain to use the robot during the next few days			0.837				
9. I know enough of the robot to make good use of it			0.709				
8. I have everything I need to make good use of the robot			0.677				
1. If I should use the robot, I would be afraid to make mistakes with it				0.877			
2. If I should use the robot, I would be afraid to break something				0.863			
22. I find the robot easy to use				0.632			
23. I think I can use the robot without any help				0.507			
4. I find the robot intimidating					0.828		
3. I find the robot scary					0.704		
24. I think I can use the robot when there is someone around to help me						0.769	
25. I think I can use the robot when I have a good manual						0.503	
40. I would trust the robot if it gave me advice							0.616
41. I would follow the advice the robot gives me							0.792
Total I-UTAUT Score	r = 0.926*	r = 0.675*	r = 0.635*	r = 0.437*	r = 0.402*	r = 0.492*	r = 0.650*

I-UTAUT, Italian version of UTAUT, questionnaire. Coefficients are sorted by size, those lower than 0.4 are not displayed.

** p < 0.001.

The final PCA proved to meet sphericity [χ^2^ (665) = 8,021.78, p < 0.001] and sampling adequacy (KMO = 0.85) assumptions.

Results of the first pilot SEM did not show a good fit for the 37 items modelled in terms of seven factors: MLχ^2^ (608) = 1,398.76; *p* < 0.001; RMSEA = 0.09; CFI = 0.754. The analysis of modification indices (MIs) indicated that the error terms of some of the items were significantly correlated: i.e., items 28 and 27 (MI = 107.42); items 34 and 33 (MI = 61.87); items 18 and 19 (MI = 54.00); items 22 and 23 (MI = 41.84); items 1 and 2 (MI = 41.59); items 32 and 31 (MI = 37.40); items 35 and 37 (MI = 32.51); items 35 and 36 (MI = 25.59); items 5 and 7 (MI = 23.38); items 36 and 37 (MI = 20.74); items 18 and 32 (MI = 19.74); items 6 and 7 (MI = 13.80); items 19 and 31 (MI = 13.61); items 6 and 5 (MI = 13.38); items 30 and 3 (MI = 12.97); items 19 and 32 (MI = 11.74); items 35 and 1 (MI = 11.43); items 18 and 31 (MI = 11.36); items 28 and 9 (MI = 11.10); items 1 and 22 (MI = 10.56); items 9 and 8 (MI = 10.48); items 27 and 36 (MI = 10.48); items 28 and 35 (MI = 10.43); items 19 and 33 (MI = 10.40) and items 18 and 33 (MI = 10.29). Thus, these additional paths were included in the model that was considered as the new 37-item seven-factor model and the fit of the model was tested again. Results of this pilot SEM showed an adequate fit for the corrected model that considered all the significant paths between items, MLχ^2^ (583) = 932.50; *p* = 0.081; RMSEA = 0.06; CFI = 0.901. The standardized item saturations ranged from 0.999 to 0.433 for the first factor; from 0.999 to 0.470 for the second factor; from 0.998 to 0.437 for the third factor; from 0.999 to 0.227 for the fourth factor; from 0.999 to 0.675 for the fifth factor; from 0.800 to 0.234 for the sixth factor and from 0.997 to 0.722 for the seventh factor.

Cronbach’s alpha value of the 37-item I-UTAUT was of 0.94. Item-total correlations ranged from >0.2 to 0.7 for all items except for item 1; after removing item 1 the value of Cronbach’s alpha increased from 0.94 to 0.942. However, since the removal of the item did not change substantially the value of Cronbach’s alpha, we recommended keeping it (Table 3). The average inter-item correlation was 0.301.

**TABLE 3:** Reliability of the I-UTAUT questionnaire.

	Mean ± standard deviation	Cronbach’s alpha when the item is excluded	Item-total correlation
Item 1	2.80 ± 1.08	0.942	0.138
Item 2	2.86 ± 1.12	0.942	0.242
Item 3	3.92 ± 0.95	0.940	0.345
Item 4	4.01 ± 0.096	0.940	0.410
Item 5	3.47 ± 1.00	0.937	0.695
Item 6	3.19 ± 0.99	0.938	0.609
Item 7	3.45 ± 0.94	0.938	0.580
Item 8	2.51 ± 1.01	0.939	0.437
Item 9	2.31 ± 0.93	0.939	0.510
Item 10	2.36 ± 1.11	0.939	0.549
Item 11	2.27 ± 1.08	0.938	0.594
Item 12	2.31 ± 1.14	0.938	0.556
Item 13	3.04 ± 1.06	0.938	0.630
Item 16	3.25 ± 1.12	0.937	0.716
Item 17	3.29 ± 1.11	0.936	0.768
Item 18	3.52 ± 0.96	0.936	0.782
Item 19	3.59 ± 0.91	0.936	0.804
Item 20	3.36 ± 0.93	0.939	0.518
Item 22	3.15 ± 0.89	0.938	0.590
Item 23	2.46 ± 0.89	0.939	0.473
Item 24	3.31 ± 0.96	0.941	0.243
Item 25	3.50 ± 0.89	0.939	0.538
Item 26	2.27 ± 1.09	0.938	0.559
Item 27	2.23 ± 1.12	0.937	0.682
Item 28	2.17 ± 1.04	0.937	0.656
Item 29	3.19 ± 1.00	0.937	0.727
Item 30	3.58 ± 0.83	0.937	0.764
Item 31	3.14 ± 0.96	0.937	0.743
Item 32	3.29 ± 0.97	0.936	0.786
Item 33	2.99 ± 0.97	0.938	0.557
Item 34	2.91 ± 1.04	0.938	0.609
Item 35	2.09 ± 1.00	0.939	0.539
Item 36	2.53 ± 1.07	0.939	0.493
Item 37	2.06 ± 1.05	0.939	0.483
Item 39	1.83 ± 1.04	0.940	0.344
Item 40	2.32 ± 1.07	0.938	0.641
Item 41	2.40 ± 1.08	0.938	0.608

As for the divergent validity of the questionnaire, I-UTAUT score did not correlate with MoCA score or its sub-domains (Table 4).

**TABLE 4:** Divergent validity between I-UTAUT and MoCA.

	I-UTAUT	
	r	p
MoCA total score	−0.026	0.779
*Visuospatial subscore*	−0.038	0.681
*Attention subscore*	0.014	0.880
*Language subscore*	0.022	0.812
*Memory subscore*	−0.055	0.546
*Orientation subscore*	−0.156	0.088

I-UTAUT, Italian version of UTAUT, questionnaire; MoCA, montreal cognitive assessment.

Moreover, no correlations were found between I-UTAUT and education, while a significant correlation was found between I-UTAUT and age (r = 0.207; p = 0.023).

## Discussion

The present pilot validation study revealed that the I-UTAUT did not meet the original thirteen-dimensional structure of the UTAUT with which it was conceived (i.e., ANX, ATT, FC, ITU, PAD, PENJ, PEOU, PS, PU, SI, SP, Trust, USE). Indeed, the final PCA suggests a seven-component structure describing individuals’ perception regarding certain characteristics of the robot, in particular: 1) acceptance and perceived usefulness; 2) perceived sociability and empathy; 3) individuals’ self-efficacy and intention to use the device; 4) perceived ease of use; 5) feelings of anxiety or emotional reactions; 6) need for support; 7) trusting the robot’s advice. All components were correlated with I-UTAUT total score. However, compared to the original scale, three items (i.e., 14, 15, 21) were removed due to low loadings across all components, suggesting limited alignment with the primary constructs. Additionally, item 38 was removed as it loaded solely onto a unique factor. Results of the pilot SEM with bootstrapping demonstrated an adequate fit for the 37-item seven-factor model. These findings would suggest that psychometric properties of the scale could improve after exclusion of these items and following a seven-factor structure, but further validation studies are necessary to support this change.

Furthermore, the I-UTAUT showed good data quality and acceptability as indicated by no missing value, no floor and ceiling effects. The level of reliability was high as well as the item-level reliability, although item 1 showed a lower item-total correlation. However, item 1 was retained in the scale, as its removal did not result in a substantial improvement in the overall reliability. Moreover, considering the preliminary nature of these findings, we opted to preserve the original structure of the scale to maintain its conceptual integrity. Moreover, the average inter-item correlation witnesses the internal consistency of the questionnaire.

As for divergent validity, we found not significant correlations between the I-UTAUT and MoCA, suggesting that the level of robotics technology acceptance is not influenced by cognitive abilities. This finding appeared to be in line with the study by Sorrentino et al. (2021) who showed that the usability and the user’s perception of the robotics technology was not affected by the cognitive status of the person.

In the present study, no association was found between score on I-UTAUT and years of schooling, but we observed a positive and significant correlation between I-UTAUT and age, suggesting that older adults were more likely to accept a social robot in daily life. Our results could be consistent with the study by Nomura et al. (2012) who showed that people in their twenties, who had experienced humanoid robots directly or in the media, reported higher anxiety levels toward robots than those aged 50–60. However, it is worth noting that older individuals showed a greater tendency to present themselves in a positive light, being more likely to respond to questionnaire items in socially desirable ways (Ausmees et al., 2022). This could be an issue to consider when interpreting this result.

Our study is characterized by some strengths. Indeed, it is the first one to provide data on the psychometric soundness of the Italian version of the UTAUT questionnaire, even covering a wide range of adult age (from 30 to >80 years) and balancing according to gender (see Supplementary Material S2).

Regarding limitations, it should be noted that this study provides construct validity evidence only by means of divergent validity. As a consequence, further investigations are needed to test the convergent validity of the I-UTAUT towards other measures of technology acceptance. Similarly, future studies should better examine test-retest reliability. Moreover, although PCA and pilot SEM analyses were conducted, our sample size was limited and insufficient to support a full exploratory and confirmatory factor analysis. Consequently, the findings should be considered preliminary and require further confirmation in future studies with larger samples.

In conclusion, we demonstrated that the I-UTAUT is a valid and reliable questionnaire to explain and predict human behaviour for hedonic system like social robot, and its adoption is encouraged in clinical practice and research.

## Data Availability

The raw data supporting the conclusions of this article will be made available by the authors, without undue reservation.
