# Supplementary material for: The Italian version of the unified theory of acceptance and use of technology questionnaire: a pilot validation study
Source: Front Robot AI. 2025 Feb 17;12:1371583. doi: 10.3389/frobt.2025.1371583 (PMC11872730; doi:10.3389/frobt.2025.1371583)
Supplement: Supplementary file 1 [file Supplementaryfile1.docx]

**Supplemental Material 1. Italian version of the UTAUT questionnaire (I-UTAUT)**

Nome _______________________ Cognome ___________________ Età ________________

Scolarità ___________

Per favore, cerchia la risposta che ritieni più appropriata

1. Come descriveresti la tua conoscenza generale dei computer?
2. Molto scarsa b. scarsa c. nella media d. buona e. molto buona
3. Come descriveresti la tua conoscenza di Internet?
4. Molto scarsa b. scarsa c. nella media d. buona e. molto buona
5. Quanto spesso usi Internet ogni giorno?
6. Molto scarsa b. scarsa c. nella media d. buona e. molto buona

**Versione Italiana dell’UTAUT Questionnaire**

Per favore, indica con una crocetta (x) il tuo livello di accordo o disaccordo con le seguenti affermazioni:

|  | **Totalmente in disaccordo** | **In disaccordo** | **Non so** | **D’accordo** | **Totalmente d’accordo** |
| --- | --- | --- | --- | --- | --- |
| 1. Se dovessi usare il robot, avrei paura di fare degli errori |  |  |  |  |  |
| 1. Se dovessi usare il robot, avrei paura di rompere qualcosa |  |  |  |  |  |
| 1. Trovo che il robot mi faccia paura |  |  |  |  |  |
| 1. Trovo che il robot sia minaccioso |  |  |  |  |  |
| 1. Penso che sia una buona idea usare un robot |  |  |  |  |  |
| 1. Il robot potrebbe rendere la mia vita più interessante |  |  |  |  |  |
| 1. È una buona idea usare il robot |  |  |  |  |  |
| 1. Ho tutto ciò di cui ho bisogno per poter utilizzare il robot |  |  |  |  |  |
| 1. So abbastanza del robot per farne buon uso |  |  |  |  |  |
| 1. Penso che userò il robot nei prossimi giorni |  |  |  |  |  |
| 1. Sono certo di usare il robot nei prossimi giorni |  |  |  |  |  |
| 1. Sto pianificando di usare il robot nei prossimi giorni |  |  |  |  |  |
| 1. Penso che il robot sia capace di adattarsi ai miei bisogni |  |  |  |  |  |
| 1. Penso che il robot farà solo ciò di cui ho bisogno in quel preciso momento |  |  |  |  |  |
| 1. Penso che il robot mi aiuterà quando io lo riterrò necessario |  |  |  |  |  |
| 1. Trovo piacevole che il robot mi parli |  |  |  |  |  |
| 1. Mi piace svolgere attività con il robot |  |  |  |  |  |
| 1. Penso che il robot sia piacevole |  |  |  |  |  |
| 1. Penso che il robot sia interessante |  |  |  |  |  |
| 1. Penso che il robot sia noioso |  |  |  |  |  |
| 1. Credo che imparerò presto come usare il robot |  |  |  |  |  |
| 1. Trovo che il robot sia semplice da usare |  |  |  |  |  |
| 1. Penso di poter utilizzare il robot senza alcun aiuto |  |  |  |  |  |
| 1. Penso di poter usare il robot quando c’è qualcuno che può aiutarmi |  |  |  |  |  |
| 1. Penso di poter usare il robot grazie ad un buon manuale |  |  |  |  |  |
| 1. Ritengo che il robot sia un buon compagno per fare una conversazione |  |  |  |  |  |
| 1. Trovo sia piacevole interagire con il robot |  |  |  |  |  |
| 1. Sento che il robot mi capisce |  |  |  |  |  |
| 1. Credo che il robot sia gradevole |  |  |  |  |  |
| 1. Credo che il robot mi sia utile |  |  |  |  |  |
| 1. Sarebbe utile per me avere un robot |  |  |  |  |  |
| 1. Penso che il robot possa aiutarmi in molte cose |  |  |  |  |  |
| 1. Penso che i miei familiari e le persone che mi aiutano apprezzerebbero se usassi il robot |  |  |  |  |  |
| 1. Penso che darei una buona impressione se usassi il robot |  |  |  |  |  |
| 1. Interagendo con il robot, mi è sembrato di parlare con una persona vera |  |  |  |  |  |
| 1. Talvolta mi è sembrato che il robot mi guardasse davvero |  |  |  |  |  |
| 1. Riesco ad immaginare il robot come una creatura vivente |  |  |  |  |  |
| 1. Penso spesso che il robot non sia una persona vera |  |  |  |  |  |
| 1. Talvolta sembra che il robot abbia sentimenti veri |  |  |  |  |  |
| 1. Mi fiderei del robot se mi desse dei consigli |  |  |  |  |  |
| 1. Seguirei i consigli suggeriti dal robot |  |  |  |  |  |
