# Supplementary material for: The Italian version of the unified theory of acceptance and use of technology questionnaire: a pilot validation study
Source: Front Robot AI. 2025 Feb 17;12:1371583. doi: 10.3389/frobt.2025.1371583 (PMC11872730; doi:10.3389/frobt.2025.1371583)
Supplement: Supplementary file 2 [file Supplementaryfile2.docx]

**Supplementary Material 2. Sample stratified by age and sex**

|  | 30-39 | 40-49 | 50-59 | 60-69 | 70-79 | ≥80 | Total |
| --- | --- | --- | --- | --- | --- | --- | --- |
| Men | 12 | 12 | 7 | 12 | 5 | 5 | 53 |
| Women | 18 | 16 | 16 | 8 | 4 | 6 | 68 |
| Total | 30 | 28 | 23 | 20 | 9 | 11 | 121 |
